# Targeted replacement of human γδ TCR in mice enhances antigen-specific B cell immunity

**DOI:** 10.3389/fimmu.2026.1734493

**Published:** 2026-06-17

**Authors:** Husheem Michael, Michael Pitre, Gene W. Weng, Mikaela M. Vallas, Ellen Chen, Paul Sheiffele, Wei Weng

**Affiliations:** Cell Biology and Immunology, InGenious Targeting Laboratory, Holbrook, NY, United States

**Keywords:** antibody production, antibody secreting plasma B cell, B cells, humanized γδ TCR mice, immunization

## Abstract

The humanized (Hu) gamma delta (γδ) T cell receptor (TCR) -T1 (γδ HuTCR-T1) mouse model represents a novel and versatile platform for investigating human-like immune responses *in vivo*. In this study, we assessed the adaptive and humoral immune responses of γδ HuTCR-T1 mice compared with wild-type (WT) counterparts following immunization with collagen and keratin antigens. γδ HuTCR-T1 mice exhibited markedly enhanced B cell activity, as demonstrated by a significant increase in antigen-specific antibody-secreting cells (ASCs), elevated serum titers of antigen-specific IgG, IgA, and IgE, and expansion of both plasma and memory B cell populations within spleen and blood tissues. In addition to the amplified B cell responses, γδ HuTCR-T1 mice displayed a distinct and dynamic cytokine profile, characterized by increased production of interleukin (IL)-4, IL-6, IL-10, IL-17, TGFβ, IFNγ, and TNFα, reflecting a balanced pro- and anti-inflammatory immune landscape. Importantly, serum levels of anti-ANA antibodies remained below the detection threshold. Collectively, these findings demonstrate that human γδ T cells modulate adaptive and humoral immunity through both direct cellular interactions and cytokine-driven mechanisms that promote B cell maturation, activation, and class switching. This work advances our understanding of γδ T cells as critical regulators of immune homeostasis and highlights the γδ HuTCR-T1 model as a valuable translational resource for preclinical studies.

## Introduction

The immune system is a highly complex network of cells and molecular pathways evolved to defend the host against infections and diseases. Among its central components are B and T cells, each performing distinct but interconnected roles. B cells are the essential effectors of an adaptive immune system and their functions that include antibody production, antigen presentation, and the generation of immunological memory (1). A specialized subset of T cells, including gamma delta (γδ) T cells, contribute to immune surveillance and occupies a niche at the interface of innate and adaptive immunity. Although γδ T cells are less abundant than their αβ T cell counterparts, they play critical roles in antibody production, early immune responses, and tumor surveillance (2–4).

The interaction between B cells and γδ T cells remains a relatively underexplored area with substantial implications for immunotherapy and vaccine development (3). γδ T cells provide “innate help” to immature B cells, facilitating their maturation into antibody-producing cells, particularly within the spleen. This support is mediated through direct cell-to-cell and cytokine signaling, influencing not only the quantity but also the specificity and quality of humoral response (3). Understanding the γδ T cell-B cell interaction could may lead to new therapeutic strategies for diseases by targeting γδ T cells to modulate B cell responses (3).

Humanized (Hu) mice bridge the gap between traditional animal models and human clinical studies. By incorporating human immune system components, these models provide a more physiologically relevant platform for studying human immune dynamics and improve the translational value of preclinical findings (5–7).

Our laboratory has generated humanized γδ T cell receptor (TCR) mice that are genetically engineered to express human γδ TCR components allowing for more relevant studies of human immune responses (8). Our recent study showed that γδ HuTCR-T1 mice exhibited a significantly higher percentage of B cells in the spleen (9). Thus, these mice can serve as invaluable tools in understanding the interactions between antigens and B cell immune responses.

For this study collagen and keratin were used as immunogens to explore B cell immune responses, leveraging their known immunogenic properties and clinical relevance. Collagen is implicated in various diseases and tissue regeneration processes (10), while keratin is important in dermatological conditions, tissue repair, and its ability to elicit robust immune responses (11). In this study, we compared B cell immune responses in γδ HuTCR-T1 and wild-type (WT) mice following immunization with collagen and keratin to investigate how humanized γδ TCR signaling influences B cell responses. By examining these immunogens, we aim to gain insight how γδ HuTCR-T1 mice modulate humoral immunity and to evaluate the potential utility of this model as a platform for studying human-relevant immune responses.

## Materials and methods

### Generation of gamma delta humanized T cell receptor-T1 mice

All animal experiments were conducted in compliance with the Institutional Animal Care and Use Committee (IACUC) protocol (# 2023-1005) approved by the inGenious Protocol Committee and adhered to the guidelines of the American Veterinary Medical Association. Mice were housed in groups of three to four per cage under specific pathogen-free conditions with unrestricted access to food and water. γδ HuTCR-T1 mice were generated in house using the Ingenious TruHumanization platform (8, 9, 12).

### Immunization, samples collection, and isolation of mononuclear cells

Male and female γδ HuTCR-T1 mice (6–8 weeks old) and genetic background–matched 129/C57/B6 wild-type (WT) controls were immunized via intraperitoneal injection with 30 μg per mouse per dose of either human keratin (epidermis; Sigma-Aldrich, cat. no. K0253) or human type I collagen (skin; Human Biologics, SKU: SCS1), emulsified in complete Freund’s adjuvant (Sigma-Aldrich, cat. no. F5881). Booster immunizations with the antigens were emulsified in incomplete Freund’s adjuvant (Sigma Aldrich, cat. # F5506) and were administered weekly for four weeks. After three days, mice were euthanized using a gradual-fill carbon dioxide inhalation method at a flow rate of 5 L/min, corresponding to 30–70% chamber volume displacement per minute. All the procedures were performed in accordance with institutional and ethical guidelines, tissues including blood and spleen were collected. Single-cell suspensions and mononuclear cells (MNCs) were prepared as described previously (9, 12).

### Antibody ELISA assay

Antibody titers were measured using an enzyme-linked immunosorbent assay (ELISA) performed with minor modifications as outlined previously (13–19). Briefly, keratin or collagen (5 µg/ml) was diluted in a coating buffer (e.g., carbonate-bicarbonate buffer, pH 9.6), 100 µL of the antigen solution was added to each well of the ELISA plate (NUNC, Thermo Fisher Scientific, cat. # 442404) followed by overnight incubation at 4 °C. The coating solution was discarded and plate was washed five times with wash buffer (e.g., PBS with 0.05% Tween-20), blocked with skimmed milk (2%) prepared in PBS, and incubated for an hour at room temperature (RT). After washing, the serum samples (50 μl/well) were added in duplicates, and incubated for two hours at RT. After washing, 100 µL of goat anti-mouse HRP-conjugated IgG3 (1:1000, Jackson Immuno Research Laboratories Inc, code # 115-035-209); - IgM (1:1000, SeraCare Inc, cat. # 5220-0356); - IgG (1:1000, SeraCare Inc. cat. # 5220-0355); - IgG1 (1:1000, Southern Biotech. cat. # 1071-05); - IgGb (1:1000, Southern Biotech. cat. # 1091-05); IgGc (1:1000, Southern Biotech. cat. # 1078-05); - IgA (1:1000, Southern Biotech. cat. # 1040-05); and – IgE (1:1000, Invitrogen Inc, SA5-10263) were prepared in PBS, added, and incubated for an hour at RT. Antibody binding was detected using tetramethylbenzidine (TMB) substrate (SeraCare Inc, cat. # 5120-0053), and the reaction was stopped with 2N sulfuric acid. Absorbance was measured at 450 nm, and antibody titers were calculated by applying the following formula:

Cut-off = average absorbance of the negative control + 3 × standard deviations of the negative control. Anti-nuclear antibody (ANA) ELISA assay was performed according to the manufacturer’s instructions (Alpha Diagnostics International, Cat. # 5210).

### ELISPOT (antibody-secreting cells) assay

ASCs/ELISPOT assays were performed as previously described (13–19). Briefly, 96-well ELISPOT plates were coated overnight at 4 °C with keratin or collagen at the concentration of 5 µg/ml (diluted in carbonate buffer) and blocked with 2% skimmed milk diluted in PBS for an hour at RT. MNCs were plated at the concentration of 5 × 10^5^ per ml, centrifuged for five minutes at 500× RPM and cultured overnight at 37 °C. Media were collected and stored at -20 °C for later analysis. Spots were detected using goat anti-mouse HRP-conjugated IgG_3_, followed by detection with TMB substrate (SeraCare Inc, cat. # 5120-0053) containing a membrane enhancer (SeraCare Inc, cat. # 5420-0026). Plates were washed extensively between the steps with PBS. ASCs were quantified using a light microscope. Background spots from unstimulated control wells were subtracted from antigen-stimulated conditions.

### Flow cytometry analysis

Murine blood and splenic MNCs were isolated under sterile conditions and processed into single-cell suspensions by mechanical dissociation, followed by red blood cell lysis using ACK buffer. Cells were washed and resuspended in staining buffer (PBS containing 0.5% BSA), and viability was assessed prior to staining. To minimize non-specific binding, cells were incubated with anti-mouse CD16/CD32 Fc receptor blocking antibody, followed by staining with a fixable viability dye to exclude dead cells. Anti-mouse antibodies for flow cytometry were purchased from Biolegend Inc., CA. Surface staining was performed using fluorochrome-conjugated antibodies against CD27 (APC/Cy7, cat. # 124225), CD138 (Syndecan-1, APC, cat. # 142505), CD267 (TACI, PE, cat. # 100214), and CD44 (FITC, cat. # 103005) for 15 minutes at 4 °C in the dark. After washing, samples were acquired on a Novocyte flow cytometer. Compensation was performed using single-stained controls, and fluorescence minus one (FMO) controls were included for CD138, CD267, and CD44 to accurately define gating boundaries. Appropriate isotype-matched controls were included in all analyzes. Data acquisition was performed by collecting 50,000 events per sample using Novocyte^®^ Flow Cytometer (ACEA Biosciences Inc, San Diego, CA, USA) and data were analyzed using Novo Express^®^ software. Cells were sequentially gated as lymphocytes based on forward and side scatter, singlets, and live cells, followed by exclusion of CD3^+^ T cells. Antibody-secreting plasma B cells were identified as CD138^+^CD267^+^CD44^+^ cells. The combination of CD138^+^CD267^+^CD44^+^ provides a more comprehensive and specific phenotypic definition of antibody-secreting cells, capturing both early plasmablasts and mature plasma cells while minimizing contamination from non-activated B cell subsets.

### Cytokine determination from serum samples

Cytokine levels in serum samples were measured using commercially available ELISA kits, according to manufacturer’s instructions. The following anti-mouse ELISA kits were used [interleukin/(IL)-4 (cat #88-7044-86), IL-6 (cat. # 88-7064-88), IL-10 (cat. # 88-7105-88), tumor necrosis factor alpha/(TNFα) (cat. # 88-7324-88), IL-17A/F (cat. # 88-8711-22), interferon gamma/(IFNγ) (cat. # 88-7314-88), and TGFβ (cat. # 88-8350-88)]. All purchased from Thermo Fisher Scientific Inc.

### Statistical analysis

All statistical analyzes were performed using GraphPad Prism version 8.0.2 (GraphPad Software, Inc., La Jolla, CA). Log_10_-transformed isotype ELISA antibody titers were analyzed using one-way analysis of variance (ANOVA) followed by Duncan’s multiple-range test for *post hoc* comparison. Correlation analysis was performed using Spearman’s nonparametric correlation method. Data are presented as the mean numbers of antigen-specific antibody-secreting cells per 5 × 10^5^ MNCs and using a nonparametric *t*-test (Mann-Whitney). Error bars represent the standard errors of the means (SEM, *n* = 7-10), and a *P* value of <0.05 was considered statistically significant.

## Results

### Gamma delta humanized T cell receptor mice show enhanced antibody-secreting cells response and serum antibody titers following keratin and collagen immunizations

**Figure 1:**
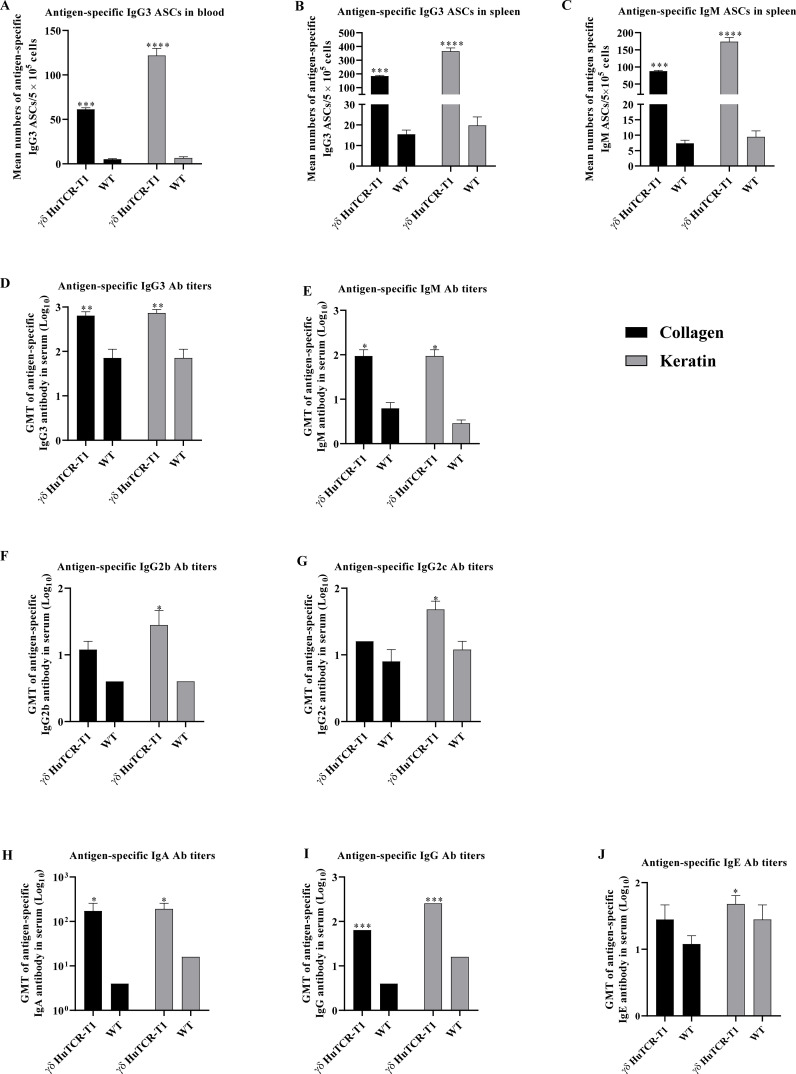
Humanized (Hu) gamma delta (γδ) T cell receptor (TCR)-T1 mice exhibit elevated antibody-secreting cells (ASCs) responses and increased serum antibody (Ab) levels after keratin and collagen immunization. Mean numbers of antigen-specific IgG3 ASCs in blood **(A)**, mean numbers of antigen-specific IgG3 **(B)** and antigen-specific IgM ASCs in spleen **(C)**. Data represent the mean numbers of antigen-specific ASCs per 5 × 10^5^ mononuclear cells (MNCs) and were analyzed using a nonparametric *t-*test (Mann-Whitney). Geometric mean titers (GMT) of antigens-specific IgG3 **(D)**, -IgM **(E)**, -IgG2b **(F)**, -IgG2c **(G)**, -IgA **(H)**, -IgG **(I)**, and -IgE **(J)** antibody (Ab) titers in serum. Log_10_-transformed isotype ELISA Ab titers were analyzed using one-way analysis of variance (ANOVA) followed by Duncan’s multiple-range test. Data are shown as means ± SEM (*n* = 7-11) for the γδ versus the wild-type (WT) mice and significant differences are indicated as *p < 0.05, **p < 0.01, ***p < 0.001, ****p < 0.0001.

This study assessed the immune responses to keratin and collagen immunization in γδ humanized T cell receptor- T1 (γδ HuTCR-T1) mice compared to wild-type (WT) controls. Four weeks post immunization with keratin and collagen, γδ HuTCR-T1 mice showed a significantly higher number of antigen-specific IgG3 antibody secreting cells (ASCs) in both peripheral blood and splenic cells compared to WT mice (**Figures 1A, B**). Notably, the numbers of keratin-specific IgG3 ASCs in blood were correlated with their splenic counterparts (*R* = 0.5, *P* = 0.02) γδ HuTCR-T1 mice (*R* = 0.4, *P* = 0.04). In addition, γδ HuTCR-T1 mice demonstrated significantly elevated numbers of antigen-specific IgM ASCs in splenic cells relative to WT controls (**Figure 1C**). Keratin-specific IgG3 ASCs in the spleen were correlated with keratin-specific IgM ASCs (*R* = 0.5, *P* = 0.03), and a similar correlation was observed for collagen-specific IgG3 ASCs and IgM ASCs of splenic cells in γδ HuTCR-T1 mice (*R* = 0.4, *P* = 0.02). Antigen-specific IgM ASCs were below the detection limit in blood cells.

Consistent with the ASCs data, serum levels of antigen-specific IgG_3_ and - IgM Ab titers were significantly elevated in γδ HuTCR-T1 mice compared with WT mice (**Figures 1D, E**). Moreover, collagen and keratin-specific IgG3 Ab titers were significantly correlated with their respective IgM Ab titers in γδ HuTCR-T1 mice (*R* = 0.2, *P* = 0.03; *R* = 0.6, *P* = 0.05, respectively).

Additionally, γδ HuTCR-T1 mice displayed increased titers of additional Ab isotypes, including antigen-specific IgG2b,c, IgA, IgG, and IgE titers when compared to WT controls (**Figures 1F–J**). Antigens-specific IgG1 Ab titers were comparable among the groups notably, while anti-ANA Ab titers were undetectable in sera (data not shown). Collectively, these findings indicate that γδ HuTCR-T1 mice have enhanced immune responses compared to WT mice, as evidenced by increased frequencies of antigen-specific ASCs and higher serum Ab titers following immunization with keratin and collagen.

### Collagen and keratin immunizations increased the frequencies of memory and antibody-secreting plasma B cells in γδ HuTCR-T1 mice

**Figure 2:**
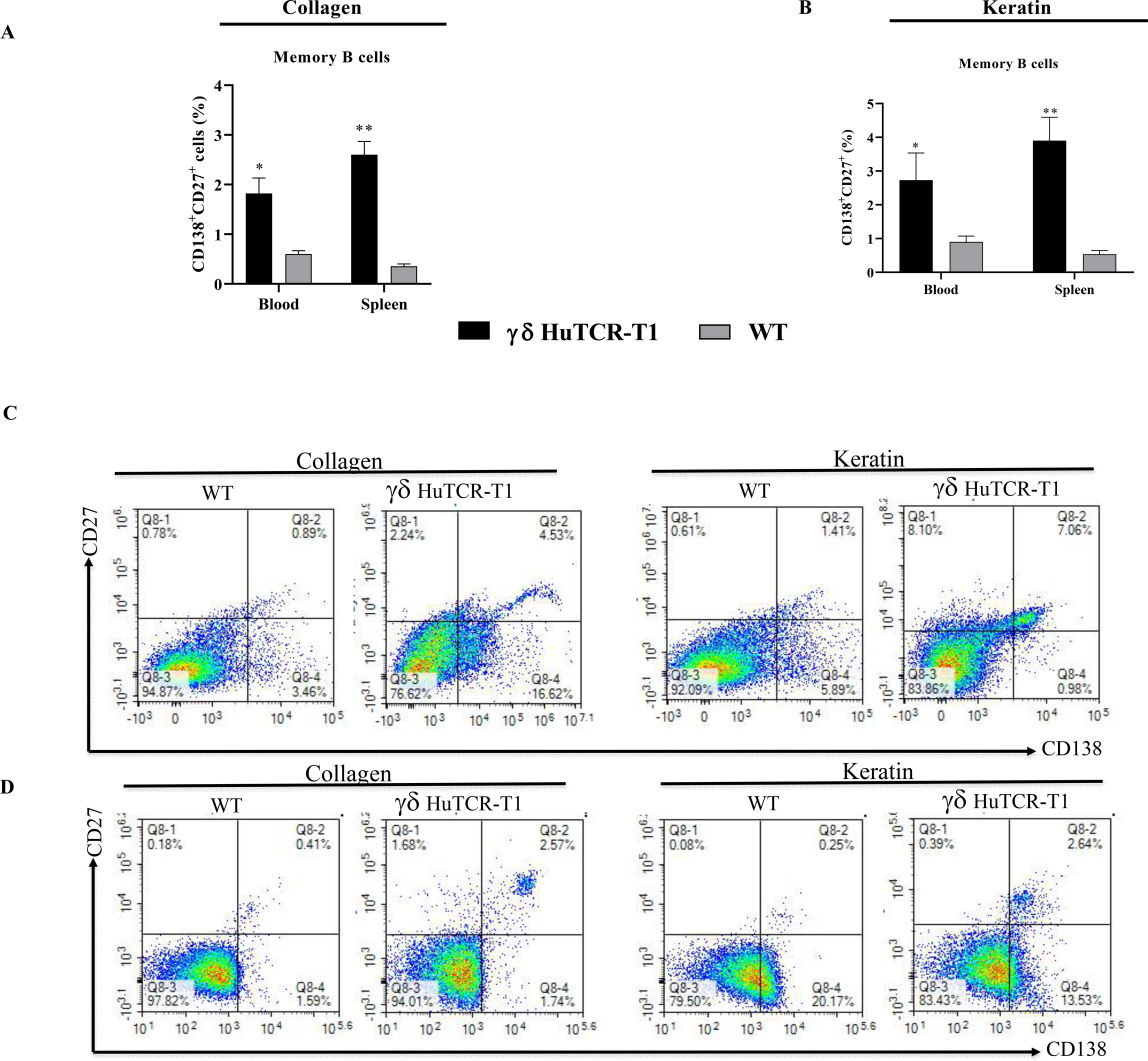
Antigens immunization increases the frequencies of memory B cells in humanized (Hu) gamma delta (γδ) T cell receptor (TCR)-T1 mice. Mean frequencies of memory (CD138^+^CD27^+^) B cells among the blood and splenic mononuclear cells (MNCs) in collagen and keratin **(A–D)** immunized mice. Flow cytometry analysis showing dot plots for blood **(C)** and splenic MNCs **(D)**. Data are shown as means ± SEM (*n* = 7-11) and were analyzed using one-way analysis of variance (ANOVA) followed by Duncan’s multiple-range test for the γδ versus the wild-type (WT) mice and significant differences are indicated as *p < 0.05, **p < 0.01.

Antigen immunization (CD138+CD27+) led to a significant increase in the proportions of memory B cells in spleen and blood MNCs of γδ HuTCR-T1 mice compared with WT controls (**Figures 2A–D**; **Supplementary Figure 1**). This increase in B cell subsets coincided with enhanced antigen-specific ASCs and higher serum Ab titers.

**Figure 3:**
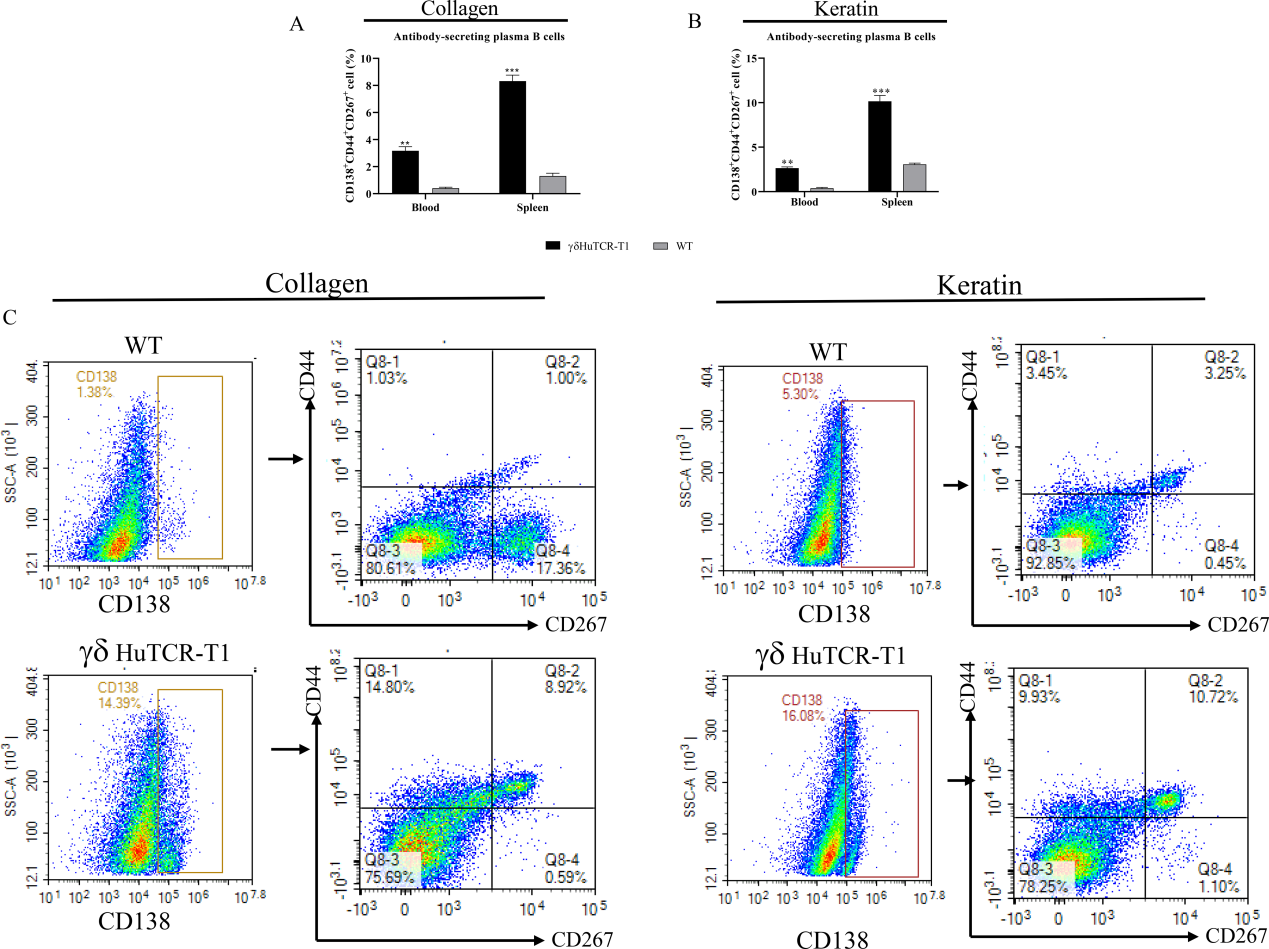
Antigens immunization elevates the frequencies of antibody-secreting plasma B cells in humanized (Hu) gamma delta (γδ) T cell receptor (TCR) mice. Mean frequencies of antibody-secreting plasma B cells (CD138^+^CD267^+^CD44^+^) in splenic and blood mononuclear cells (MNCs) of collagen and keratin immunized mice **(A–C)**. Flow cytometry analysis showing dot plots for splenic MNCs **(C)**. Data are shown as means ± SEM (*n* = 7-11) and were analyzed using one-way analysis of variance (ANOVA) followed by Duncan’s multiple-range test for the γδ versus the wild-type (WT) mice and significant differences are indicated as **p < 0.01, ***p < 0.001.

Antibody-secreting plasma B cells were identified as CD138^+^CD267^+^CD44^+^ cells. Similarly, the percentage of these cells was significantly higher in splenic and blood MNCs of γδ HuTCR-T1 mice following immunization than in WT mice (**Figures 3A–C**; **Supplementary Figure 2**). Together, these results demonstrated that γδ HuTCR-T1 mice exhibit a heightened antibody-secreting plasma B cell response following immunization, suggesting an enhanced capacity to mount antigen-specific humoral immunity.

### Keratin and collagen immunizations enhanced the cytokines levels in serum of γδ HuTCR-T1 mice

**Figure 4:**
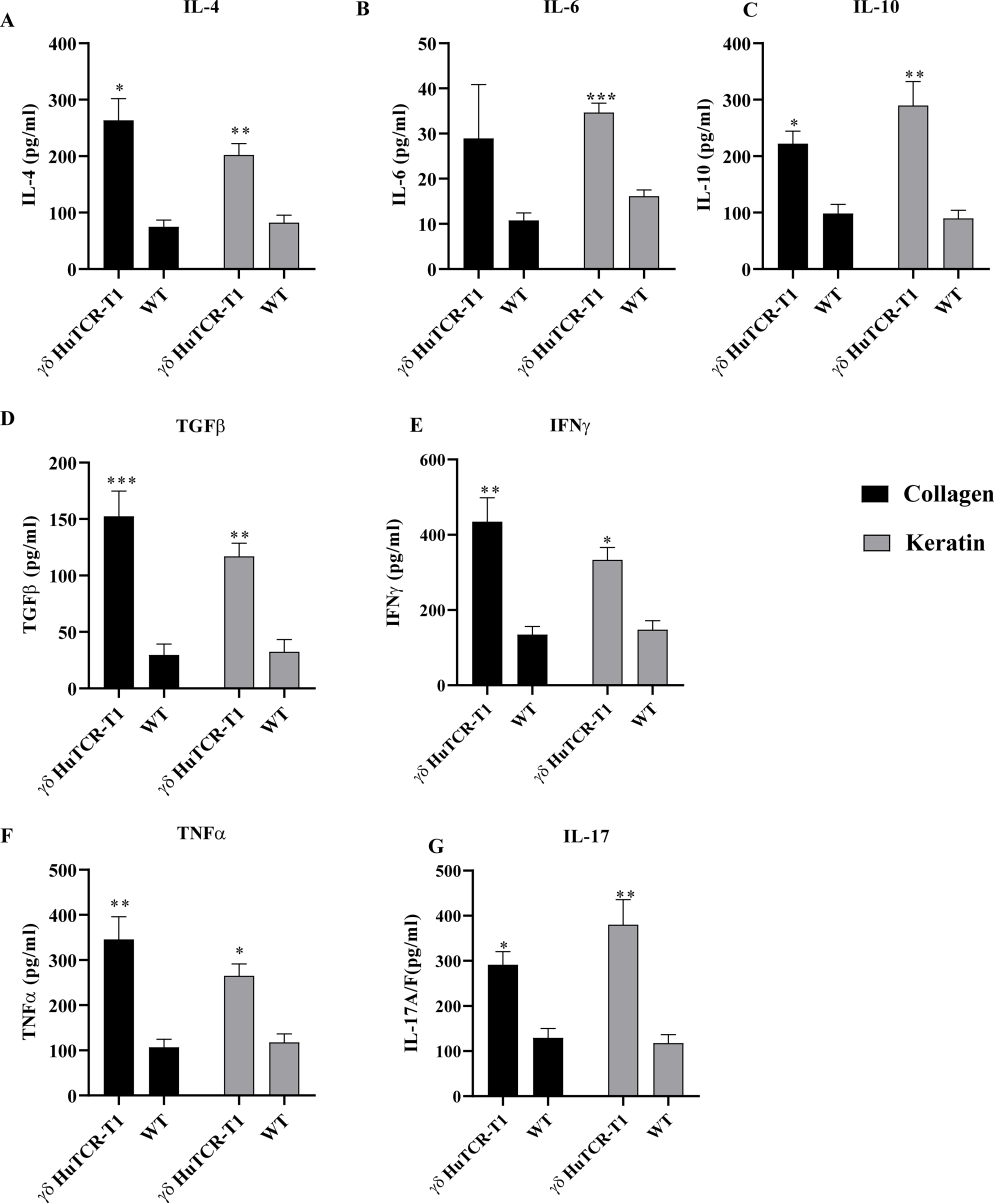
Antigens immunization increases the levels of immunoregulatory and pro-inflammatory cytokines in the serum of humanized (Hu) gamma delta (γδ) T cell receptor (TCR) mice. Levels of immunoregulatory [(e.g., interleukin (IL)-4 **(A)**, IL-6 **(B)**, IL-10 **(C)** and TGFβ **(D)**] and pro-inflammatory [(e.g., IFNγ **(E)**, TNFα **(F)**, and IL-17 **(G)**] cytokines in serum. Levels of cytokines were determined by ELISA assay. Data are shown as means ± SEM (*n* = 7-11) and were analyzed using one-way analysis of variance (ANOVA) followed by Duncan’s multiple-range test for the γδ versus the wild-type (WT) mice and significant differences are indicated as *p < 0.05, **p < 0.01, ***p < 0.001.

To assess immunoregulatory and pro-inflammatory responses elicited by antigen immunization, we measured serum cytokine concentrations by ELISA assay. Keratin and collagen immunizations significantly increased levels of key immunoregulatory cytokines, including IL-4, IL-10, and TGFβ (**Figures 4A–D**), as well as pro-inflammatory cytokines such as IFNγ, TNFα, IL-6, and IL-17 (**Figures 4E–G**). These findings indicate that immunization with keratin and collagen induces a robust and mixed cytokine milieu in γδ HuTCR-T1 mice, characterized by concurrent upregulation of immunoregulatory and pro-inflammatory mediators. However, the functional consequences of these cytokine changes on immune regulation and B cell responses require further experimental validation.

## Discussion

The immune system’s ability to recognize and respond to diverse antigens and pathogens relies on a highly coordinated interplay among multiple immune cell subsets, with B cells and T cells serving as central mediators. Within this framework, γδ T cells have emerged as key modulators bridging innate and adaptive immunity, capable of rapidly sensing stress signals and orchestrating downstream immune responses. However, the complexity of human γδ T cell biology has remained challenging to study due to the lack of physiologically relevant *in vivo* models. To address this gap, we developed a humanized γδ TCR mouse model, termed γδ HuTCR-T1, which harbors a fully functional human immune system. In this study, we utilized this model to investigate B-cell immune responses and balanced cytokine profiles, providing novel insights into the regulatory roles of γδ T cells in shaping adaptive immunity.

To achieve humanization of the mouse γδ TCR, we performed extensive genetic engineering on both the mouse γ and δ loci (8). While it is challenging to precisely pinpoint which specific genetic alterations are directly responsible for the observed B-cell immune responses, we propose several hypotheses that may help explain the experimental outcomes observed in our γδ HuTCR-T1 mice.

In the normal mouse spleen, the largest population of γδ T cells expresses Vγ1, followed by Vγ4 (20). Extensive studies have shown that these subsets play a critical role in regulating B-cell immune responses and often exert opposing effects on host immunity, influencing responses to infection, allergic inflammation, and malignancy (21–24). This functional opposition suggests that Vγ1 and Vγ4 subsets act in a counterbalancing manner to modify overall immune homeostasis (25). Insights from Vγ1^-^/^-^, Vγ4^-^/^-^, and Vδ^-^/^-^ mouse models further highlight the importance of γδ T cells in antibody regulation: deletion of all γδ T cells (Vδ^-^/^-^) caused a **~**2-fold reduction in total serum Ig compared to WT, while Vγ1^-^/^-^ mice exhibited a 6-fold decrease, and conversely, deletion of Vγ4 or Vγ6 led to a 4-fold Ig increase. These changes extended across multiple Ig subclasses (IgM, IgG1, IgG2b, IgE) and autoantibodies, with Vγ1^-^/^-^ mice showing a 2-fold reduction in autoantibody production, whereas Vγ4^-^/^-^ and Vγ6^-^/^-^ mice displayed a striking 10-15-fold increase (26). Importantly, these findings were obtained in immune-unprimed mice, underscoring the intrinsic role of γδ T cells in maintaining baseline B-cell activity and antibody homeostasis. To investigate human γδ T cell biology, we engineered the γδ HuTCR-T1 mouse model, replacing the endogenous mouse Vγ1 gene with human Vγ9, Vγ10, and Vγ11 segments; notably, human Vγ9 was inserted downstream of the mouse Vγ1 promoter, allowing its expression to mimic native Vγ1, while Vγ10 and Vγ11 retained their own human promoters. On the δ locus, we replaced mouse Vδ4, along with associated D and J segments, with human Vδ1-Vδ8 variable regions and corresponding human D and J elements, enabling γδ HuTCR-T1 mice to generate fully human γδ TCRs with physiologically relevant human CDR3 sequences (8). Baseline measurements in non-immunized HuTCR-T1 and WT mice had been performed. These data demonstrate that the baseline levels of the parameters analyzed including ASCs, cytokines, and B cell subsets were below the detection limit in HuTCR-T1 and WT mice. Following immunization, the enhanced responses observed in HuTCR-T1 mice are therefore attributable to the immunization rather than pre-existing elevated baseline levels (9). In addition, it suggested that the introduction of human γδ TCR elements compensates for the deletion of mouse Vγ1, restoring Ig regulation despite the sixfold Ig reduction observed in Vγ1^-^/^-^ mice (27). This restoration is unexpected, given the minimal sequence homology between human and mouse γδ TCRs and the difficulty of aligning Vγ and Vδ gene segments across species. One plausible explanation is that the functional interaction between γδ TCRs and their ligands or binding partners which influences B-cell activation and Ig production is evolutionarily conserved despite sequence divergence. Supporting this idea, previous studies suggested that human Vγ9Vδ2 T cells exhibit functional similarities to mouse Vγ1 T cells in responding to specific antigens (28, 29). Consistent with this, our γδ HuTCR-T1 mice, which carry human Vγ9 and Vδ2 elements, may effectively recapitulate Vγ1-like immune regulation within a humanized immune context (8).

Compared to WT controls, γδ HuTCR-T1 mice showed increased frequencies of ASCs, elevated titers of antigen-specific IgA, IgE, IgG, IgG_2_, and IgG_3_ Ab titers and an expansion of plasma and memory B cell. These findings suggest that human γδ TCR expression may have promoted stronger B cell activation, class switching, and Ab production, potentially through cytokine that may have mediated help or direct cell-cell interactions (30–33). The enhanced isotypes distribution may indicates roles in mucosal, allergic, and inflammatory immunity, consistent with a more mature and effective humoral immune response than in WT mice, which may have retained a higher proportion of naïve B cells indicating these mice are less mature, less effective, and less adapted to antigens exposure (34–36). In humans, protein-based immunization (e.g., influenza, tetanus, or hepatitis B vaccines) typically induces robust germinal center reactions, expansion of ASCs, and the generation of long-lived memory B cells and antibody-secreting plasma B cells, similar to what we observed in γδ HuTCR-T1 mice (37, 38). Therefore, our findings indicate that the presence of human γδ TCRs significantly amplifies the activity of B cells upon antigen exposure, suggesting it may have a promising avenue for preclinical research (39–41).

The robust adaptive response was accompanied by elevated serum levels of immunoregulatory and pro-inflammatory cytokines were observed in γδ HuTCR-T1 mice. This suggest that γδ TCR signaling plays a critical role in mixed cytokine response that may modulate B cell responses, offering new insights into the interplay between γδ T cells and B cells in the context of immunization (3, 31, 42–47). Our results are consistent with previous studies showing finding that γδ T cells secrete IL-4, TGFβ, and IL-10 can help B cells to differentiate into plasma and memory B cells and Abs production (27, 31, 48–52).

In addition, γδ T cells have been reported in previous studies to influence humoral immunity through multiple mechanisms, including cytokine production (e.g., IL-4, IL-10, IL-21) and modulation of T follicular helper cell differentiation, which in turn supports B cell activation and immunoglobulin production (48, 53). Moreover, γδT-cells express key mediators that interacts with B-cells, including inducible costimulatory molecule, chemokine receptor 5, programmed cell death-1 protein, and CD40L (54, 55). In these contexts, it is plausible that γδHuTCR-T1 cells may contribute to enhanced antibody responses either directly through interactions with B cells or indirectly via shaping the helper T cell environment. The definitive demonstration of direct B cell effect would require additional functional experiments, such as co-culture assays, cytokine blockade, or adoptive transfer studies. Additionally, humanized γδ TCR is expressed in murine γδ T cells may be under the control of endogenous regulatory elements. Thus, the majority of murine γδ T cells in these mice express the human γδ TCR, while other immune cell populations remain unmodified (9). γδ T cells are known to reside in both lymphoid and peripheral tissues (56) and the presence and proportion of γδ HuTCR-T1 expressing T cells in key tissues, including spleen, blood, lymph nodes, lung, thymus, ileum, and liver has been previously reported (9). It confirms that humanized γδ T cells are present in these tissues where they can influence immune responses through cytokine production and interactions with B and T cells. However, the observed immune changes are associated with the presence of humanized γδ TCR-expressing cells, and that further mechanistic studies (e.g., depletion or adoptive transfer approaches) will be required to definitively establish their functional role.

Notably, anti-ANA antibodies remained undetectable in sera of γδ HuTCR-T1 mice despite the robust keratin and collagen immunizations. This suggests that the augmented γδ T cells may helped in this model preferentially expanded B-cell clones specific for the immunizing antigens. The functional implications of these cytokine changes remain to be determined, and that additional mechanistic studies will be necessary to establish their biological significance.

Interestingly, TCRγδ deficient mice are associated with reduced total serum Ig levels and a reduction in anti-chromatin antibody production, along with altered immunoglobulin subclass composition characterized by reduced IgA and IgE levels suggesting a dampening of certain autoreactive B cell responses (27, 48). In contrast, γδ HuTCR-T1 mice where murine Vγ1 was replaced with human Vγ9–11 did not show reduced responses. Instead, keratin and collagen immunizations elicited stronger antigen-specific immunity than WT without inducing pathological autoantibodies. This difference may reflect intrinsic distinctions between human and murine γδ TCR repertoires, cytokine profiles, or accessory cell interactions in the humanized context. Therefore, this model offers valuable insights into the regulator role of γδ T cells in immunity and closely mimics human immune responses, making it a promising tool for advancing treatments/therapeutics for various diseases, infections, cancer (44, 57, 58).

### Limitation

While humanized γδ HuTCR-T1 mice provide a valuable model for studying enhanced B cell immune responses following immunization with collagen and keratin, several limitations must be acknowledged. First, species-specific differences which may not fully recapitulate the complexity of human immune responses. Second, smaller number of γδ double homozygous mice, which does not allow us to perform several analytical analyzes such as examining sex and age, which can impact the interpretation and application of findings. Third, the observed immunological changes are correlative and occur in the context of the humanized γδ TCR expressing environment, rather than representing direct mechanistic proof of cell-specific function. Thus, γδ T cells may influence immune responses, including cytokine-mediated effects and modulation of helper T cell responses.

### Future directions

Future research should aim to focus on elucidating the precise mechanisms by which γδ TCR humanization enhances B cell responses. Key areas (or components) include the direct interactions between γδ T cells and B cells, the role of specific cytokines, and the signaling pathways involved. Furthermore, evaluating immune responses across multiple time points and assessing long-term immunity following various immunization protocols would provide deeper insight into how humanized γδ T cells influence the magnitude and persistence of immune responses in γδ HuTCR-T1 mice.

### Conclusion

**In conclusion,** our study demonstrates that humanization of the γδ TCR enhances B cell responses to keratin and collagen immunizations as evidenced by increased antigen-specific Ab titers, elevated frequencies of plasma and memory B cells, and a cytokine profile favoring immune activation. γδ HuTCR-T1 mice thus represent a promising model for studying adaptive and humoral immunity. These findings pave the way for further exploration of γδ T cell- B cell cross talk and highlight the potential of this model for advancing preclinical research.

## Data Availability

The datasets generated for this study can be found here: https://doi.org/10.5281/zenodo.20430607.
