# Supplementary figures and images for: Targeted replacement of human γδ TCR in mice enhances antigen-specific B cell immunity
Source: Front Immunol. 2026 Jun 17;17:1734493. doi: 10.3389/fimmu.2026.1734493 (PMC13318656; doi:10.3389/fimmu.2026.1734493)

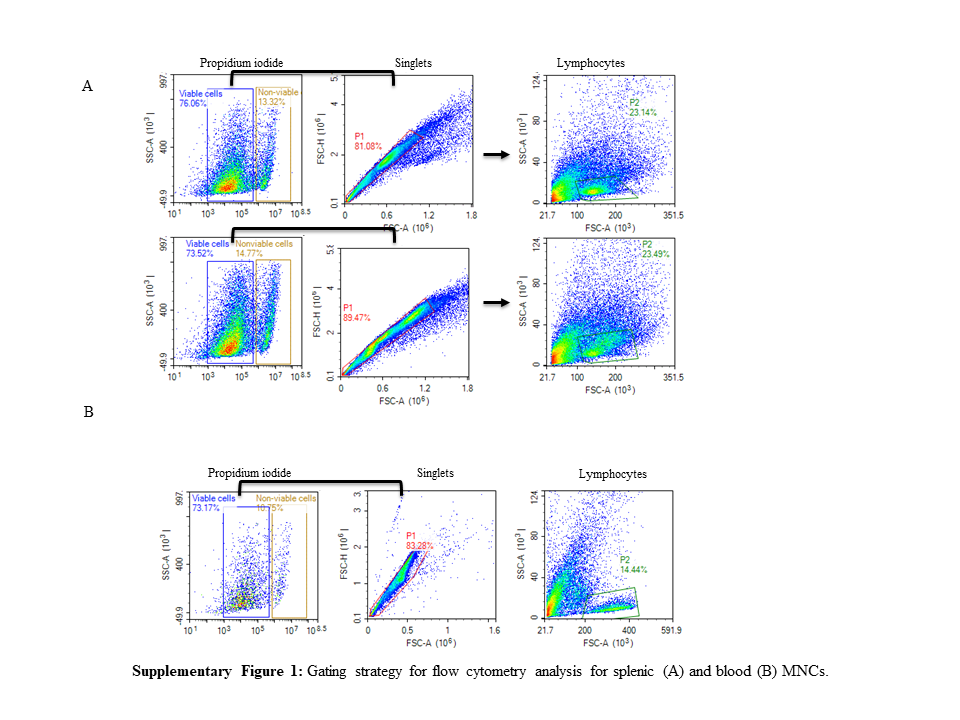

Supplement: Supplementary file 1 [file Image1.tif]

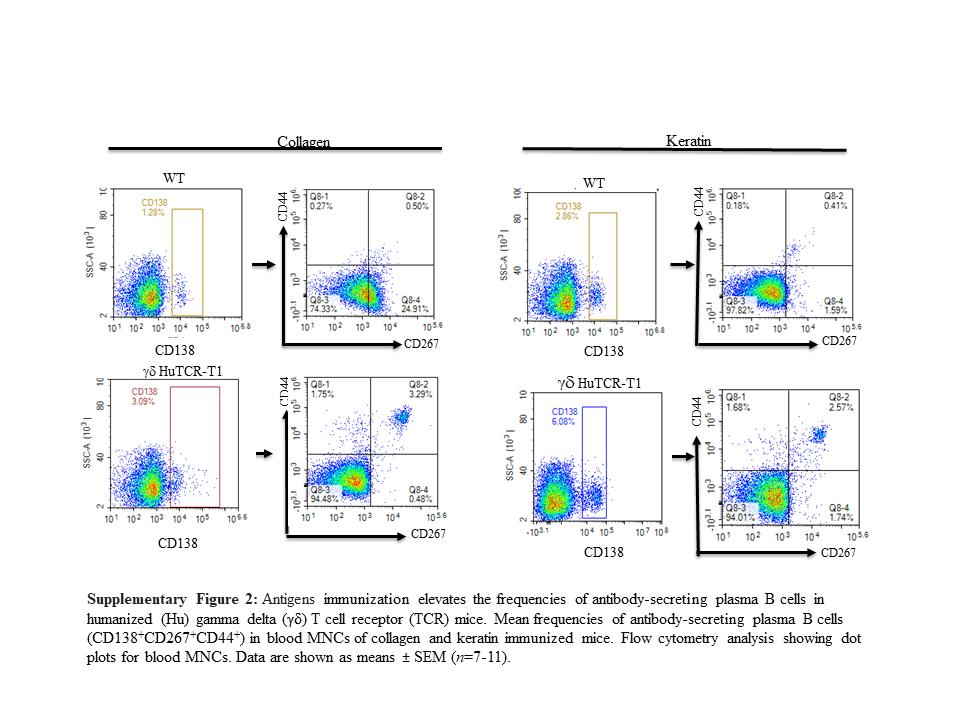

Supplement: Supplementary file 2 [file Image2.tif]
